# Decoding speech intent from non-frontal cortical areas

**DOI:** 10.1088/1741-2552/adaa20

**Published:** 2025-02-13

**Authors:** Prashanth Ravi Prakash, Tianhao Lei, Robert D Flint, Jason K Hsieh, Zachary Fitzgerald, Emily Mugler, Jessica Templer, Matthew A Goldrick, Matthew C Tate, Joshua Rosenow, Joshua Glaser, Marc W Slutzky

**Affiliations:** 1 Departments of Biomedical Engineering, Northwestern University, Chicago, IL 60611, United States of America; 2 Neurology, Northwestern University, Chicago, IL 60611, United States of America; 3 Linguistics, Northwestern University, Chicago, IL 60611, United States of America; 4 Neurosurgery, Northwestern University, Chicago, IL 60611, United States of America; 5 Neuroscience, Northwestern University, Chicago, IL 60611, United States of America; 6 Physical Medicine & Rehabilitation, Northwestern University, Chicago, IL 60611, United States of America; 7 Department of Neurosurgery, Neurological Institute, Cleveland Clinic Foundation, Cleveland, OH, United States of America

**Keywords:** speech intent, electrocorticography, brain machine interfaces, speech production, brain computer interface, aphasia, stereoencephalography

## Abstract

*Objective*. Brain machine interfaces (BMIs) that can restore speech have predominantly focused on decoding speech signals from the speech motor cortices. A few studies have shown some information outside the speech motor cortices, such as in parietal and temporal lobes, that also may be useful for BMIs. The ability to use information from outside the frontal lobe could be useful not only for people with locked-in syndrome, but also to people with frontal lobe damage, which can cause nonfluent aphasia or apraxia of speech. However, temporal and parietal lobes are predominantly involved in perceptive speech processing and comprehension. Therefore, to be able to use signals from these areas in a speech BMI, it is important to ascertain that they are related to production. Here, using intracranial recordings, we sought evidence for whether, when and where neural information related to speech intent could be found in the temporal and parietal cortices *Approach*. Using intracranial recordings, we examined neural activity across temporal and parietal cortices to identify signals associated with speech intent. We employed causal information to distinguish speech intent from resting states and other language-related processes, such as comprehension and working memory. Neural signals were analyzed for their spatial distribution and temporal dynamics to determine their relevance to speech production. *Main results*. Causal information enabled us to distinguish speech intent from resting state and other processes involved in language processing or working memory. Information related to speech intent was distributed widely across the temporal and parietal lobes, including superior temporal, medial temporal, angular, and supramarginal gyri. *Significance*. Loss of communication due to neurological diseases can be devastating. While speech BMIs have made strides in decoding speech from frontal lobe signals, our study reveals that the temporal and parietal cortices contain information about speech production intent that can be causally decoded prior to the onset of voice. This information is distributed across a large network. This information can be used to improve current speech BMIs and potentially expand the patient population for speech BMIs to include people with frontal lobe damage from stroke or traumatic brain injury.

## 1. Introduction

The loss of the ability to communicate is one of the most devastating effects of neurological diseases. For example, amyotrophic lateral sclerosis or brainstem stroke can cause anarthria and locked-in syndrome, i.e. complete paralysis [1, 2]. Brain machine interfaces (BMIs), also called brain computer interfaces, have the potential to restore communication to those afflicted with neurological disorders. In particular, BMIs that attempt to directly decode different aspects of speech, such as phonemes, speech articulator movements, or speech audio [3–7], have made great progress toward high communication rates and accuracy in the past decade, culminating in several early-phase clinical trials in paralyzed patients [1, 2, 8]. The vast majority of BMI studies have focused on decoding intended speech from frontal speech motor cortices, i.e. ventral pre-central gyrus and the inferior frontal gyrus (IFG), along with postcentral gyrus in some cases [3–7, 9–13].

A few studies have found information from outside the frontal lobe (and postcentral gyrus) about speech, including from the parietal and temporal lobes. Such information might be useful to further improve BMI performance for people with locked-in syndrome. Further, it might enable BMIs to help people with lesions in the frontal lobe, which can cause deficits such as aphasia or apraxia of speech. For such people, no frontal lobe signals would be available to control a BMI. However, the extent to which signals from parietal and temporal lobes are related to production is unclear. These areas are involved in comprehension, as well as production—a fact recognized in aphasic patients as early as the 19th century by Wernicke. More recent studies linked lesions restricted to the superior temporal gyrus (STG) and underlying white matter to conduction aphasia, which consists of inability to repeat and paraphasic errors [14, 15]. In perceptive speech processing, imaging and intracranial recordings showed that parietal and temporal areas carry information about semantics [16–18], lexical selection [19], phonology [20], and syntax [21]. There is some fMRI [22] and magnetoencephalographic [23] evidence suggestive of phonological encoding in STG during production; however, these studies were not designed to clearly separate production from perception. Further, there is limited intracranial evidence for encoding of speech production in the temporal and parietal lobes. Since these cortices are highly involved in speech perception and comprehension, it is important to distinguish whether information is related to production or perception (including comprehension) when designing a BMI. For example, in a conversation, the BMI would need to distinguish between the user’s intended speech and perceived speech that was produced by other conversants. It is of paramount importance to not be decoding the user’s thoughts that are not intended to be spoken aloud, both for that practical reason (because they are not intended to be spoken) and for the ethical problems this could incur. Thus, it is important to determine the extent of information about intent to produce speech in non-frontal areas. Intracranial decoding evidence from these areas for phonemes [20, 24], semantics [25, 26], and sentences [27, 28] has been found. However, only two studies [26, 28] made attempt to separate information related to production from information related to perception and comprehension; the analyses in these studies did not directly address the question. Thus, it remains unclear when and how much information related to production, particularly motor intent, is present in these areas.

Here, we used the high temporal resolution of electrocorticography (ECoG) and stereoelectroencephalography (sEEG) to investigate whether, when, and where parietal and temporal cortices contain information about intended speech production. We used decoders to distinguish speech motor intent (intent to vocalize) from a silent, resting state on a single-trial level, using only causal information from temporal and parietal lobes. We controlled for the possibility that we were decoding a language processing or working memory signal, rather than speech intent. We also investigated which areas of the temporal and parietal lobes had the most information about speech intent. The results demonstrate the presence of a speech intent signal in non-frontal areas.

## 2. Methods

### 2.1. Participants and electrode locations

All procedures were approved by the Institutional Review Board of Northwestern University in accordance with the principles embodied in the Declaration of Helsinki. Written informed consent for all participants was obtained prior to beginning research. All participants were over the age of 18. All participant names and personal information have been removed.

**Figure 1.**
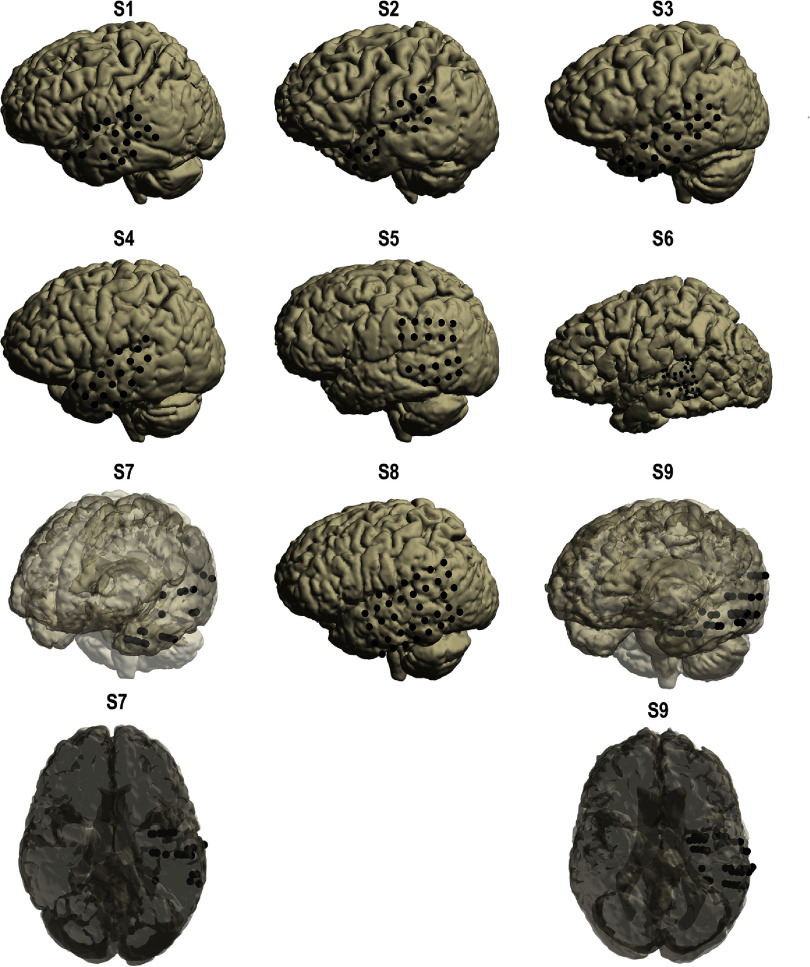
Electrode locations for every participant. Electrodes with extensive noise were removed.

We enrolled participants who required either intracranial monitoring for intractable epilepsy (4 men, 4 women) or awake craniotomies for brain tumor resection (1 male). No participants had any speech deficits. ECoG arrays and depth electrodes were placed according to clinical necessity in the participants with epilepsy, and ECoG arrays were placed over temporal and parietal lobes, at least 2 gyri away from tumor borders, as cortical exposure allowed in the participants with tumors. Participants undergoing intracranial monitoring for seizure localization received standard clinical ECoG arrays (1 cm interelectrode spacing and 2.3 mm electrode diameter) or depth electrodes (S7, S9). Intraoperative participants had higher density arrays (5 mm interelectrode spacing, 2.3 mm electrode diameter in an 8 × 8 layout) placed over temporal and/or parietal areas as allowed by the exposed surface (figure 1). Electrodes placed over the temporal and parietal lobes were manually identified using the co-registered preoperative MRI and post-operative CT scans (see *Co-registration* below). For S7 and S9, depth electrodes manually identified as being in white matter were removed.

### 2.2. Data acquisition

Audio was recorded with a unidirectional lapel microphone (Sennheiser) placed near the patient’s mouth. The audio signal was transmitted wirelessly (Caliphone) to a recording computer sampled at 48 kHz. In the intraoperative setting, stimuli were presented, and ECoG was sampled at 2 kHz and recorded on a Neuroport (Blackrock Microsystems), using BCI2000 [29]. In the participants undergoing intracranial monitoring for epilepsy, ECoG/sEEG signals were digitized at 500–2 kHz on a clinical EEG system (Nihon Kohden), determined by clinical necessity. Behavioral and ECoG recordings were synchronized using a TTL pulse recorded simultaneously on both systems.

### 2.3. Experimental protocol

**Figure 2.**
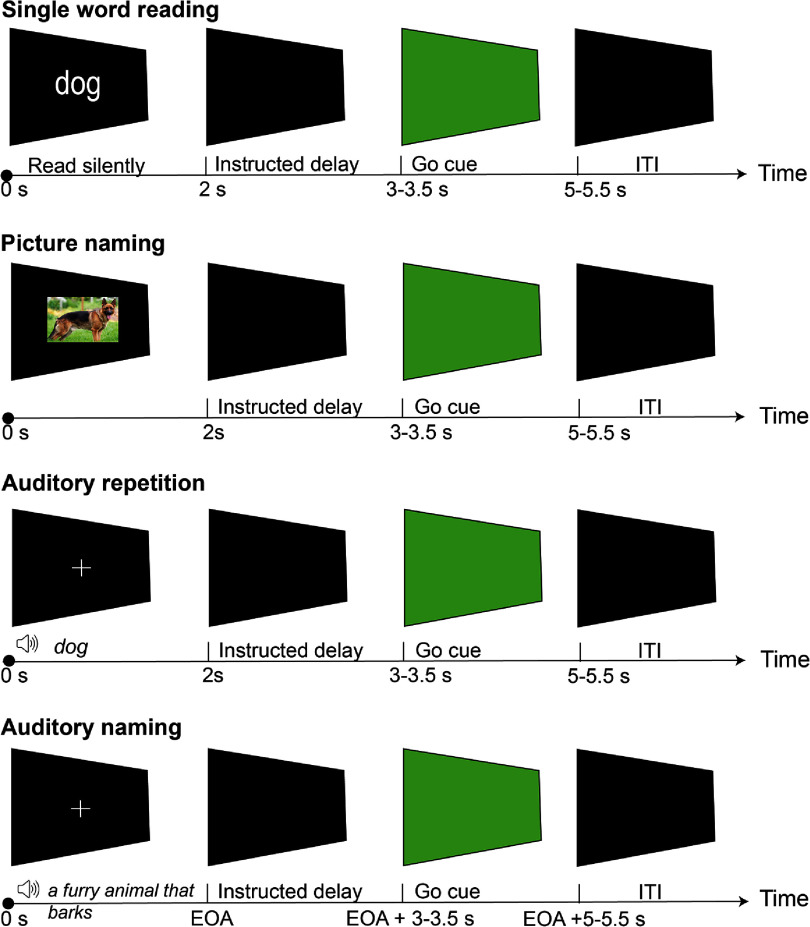
Task design schematic. In the visual tasks (reading aloud and picture naming, top two rows), the stimulus (single word or picture) was displayed, followed by a blank screen (instructed delay period), then a green screen (go cue) cueing the patient to speak the word aloud. In the auditory tasks (naming and repetition), a fixation cross was displayed on the screen as the pre-recorded audio was played on a speaker. A blank screen was presented during the intertrial interval (ITI). Timing of each interval is shown in the timeline. EOA: end of audio recording.

Participants included in this study each performed a subset of five speech tasks (table 1) divided into approximately 5-min runs. Because temporal and parietal areas are involved in multiple stages of auditory, visual, speech, and language processing, we specifically designed the different task paradigms (figure 2) to ensure that the signals being decoded were related to intent to vocalize speech (speech intent) and not other aspects of the tasks (e.g. visual or auditory processing of cues, lexical-semantic aspects of tasks, working memory). All the tasks involved participants receiving a cue that prompted them to say a monosyllabic ‘target’ word aloud, usually with an instructed delay period. Participants S4–S8 performed the single-word reading task with an instructed delay period. Here, participants were instructed to read the word silently (without movement of articulators), keep it in memory when the screen became blank (instructed delay) and articulate the word out loud when the screen turned green. This instructed delay task was designed to separate modulation in neural signals related to speech production from modulation related to reading, comprehension, and lexical selection. In addition to the delayed word-reading task, participants S7–S9 performed a picture naming task, in which they were instructed to identify the displayed picture; an auditory naming task, in which they were instructed to respond with a single word cued to a description played aloud; and an auditory repetition task, in which they were instructed to repeat a word played aloud. These tasks used the same task design, save that instead of a word, the initial cue was a picture or a pre-recorded audio description or single word. A few participants performed slightly different versions of the task. Participants S1 and S2 performed a single-word reading task without a go cue (i.e. they were instructed to speak as soon as the word was presented on the screen). Due to a technical issue the stimulus onset time was not recorded for S2. Participant S3 performed a similar single-word reading task but was instructed to say the word out loud when the word disappeared.

**Table 1.** Participant demographics and number of runs of each task performed by each participant.

Participant	Sex	Single-word reading	Auditory repetition	Picture naming	Auditory naming
S1	Male	3	0	0	0
S2	Female	3	0	0	0
S3	Female	1	0	0	0
S4	Female	1	0	0	0
S5	Male	10	0	0	0
S6	Male	2	0	0	0
S7	Male	1	4	4	0
S8	Male	3	2	6	0
S9	Female	0	2	3	2

### 2.4. Spectral analysis

**Figure 3.**
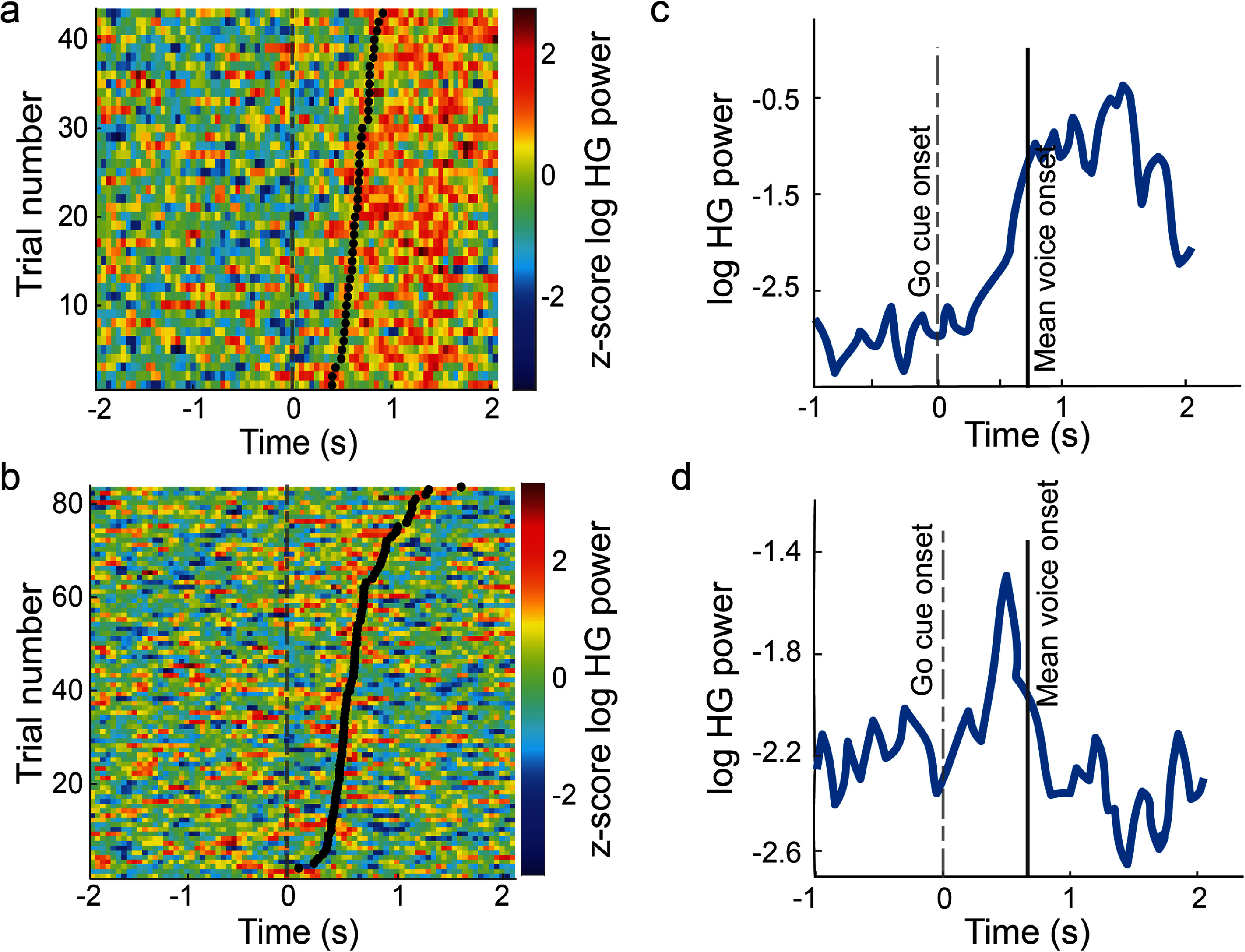
High gamma (HG) activity aligned to voice onset in representative electrodes. (a), (b) Raster plots showing HG activity (*z*-score of log HG power), one trial per row. Plots are aligned to the go cue (green dashed line at time 0). Black dots indicate voice onset time for each trial. (c), (d) Trial-averaged high gamma response. Black vertical line represents the mean voice onset time over trials. HG started prior to, and was aligned to, voice onset rather than go cue. Electrodes were located in the posterior middle temporal gyrus (a), (c) and posterior superior temporal gyrus (b), (d).

To track the evolution of high gamma activity relative to voice onset, we employed a short-time Fourier transform (512 ms width) to compute log-normalized spectral power per electrode. Spectral power was computed in 50 ms bins that was averaged across trials aligned to go cue. High gamma frequency bands were identified for each electrode within a 4-s period aligned to the go cue. Normalized (*z*-scored) raster plots were generated for individual trials, with two representative electrodes highlighted (figures 3(a) and (b)). Event-related potentials were plotted (figures 3(c) and (d)) by averaging activity across trials and frequency bands within the same time period per electrode.

### 2.5. Feature extraction and selection

The audio signals were manually labeled as speech intent or silence using audio editing software (Audacity). The raw ECoG/sEEG signals were manually examined to remove electrodes and trials with artifacts (determined by manual inspection of the time domain and frequency domain signals). Harmonics of line noise (60, 120, 180 Hz) were notch-filtered using zero-phase delay filters. To remove common-mode noise the signals were common average referenced using only the clean electrodes. High-gamma (HG) band (70–200 Hz) activity was extracted using a one-way, linear-phase FIR filter that introduced a constant delay. Since the sampling rate differed across participants (500–2000 Hz), we computed the optimal number of taps for each as $N \approx \frac{{4{f_{\text{s}}}}}{{\Delta f}}$, where *f*_s_ was the sampling rate and *Δf* was the transition bandwidth, defined as 10 Hz. The analytical amplitude was computed using the Hilbert transform [30] and the HG power was computed by squaring this signal. The delay due to filtering was $\tau = \frac{{N - 1}}{{2{f_{\text{s}}}}}$, translating to 199, 199.5, and 199.75 ms for *f*_s_ of 500, 1000 Hz and 2000 Hz, respectively. We corrected for this delay by shifting the filtered HG power backward by *τ* ms.

Mean HG power in 100 ms, non-overlapping windows was used as the feature to decode speech motor intent. For every trial, a window was assigned a class according to the respective behavioral state at that time: speech or resting state (which we call silence for brevity). We performed two types of classification analyses, one that included a single feature window (100 ms) to predict a behavioral state and another that included four consecutive feature windows (400 ms). To avoid bias due to imbalanced classes, we sampled the speech and silence features to have the same number of elements. Speech intent feature periods were defined as 100 ms (for single features) or 400 ms (for 4 features) prior to voice onset. Silence feature windows were defined as starting 1.5 s after voice offset for a word (1 or 4 windows of 100 ms, for the single and 4-feature analyses). In the participants that performed the auditory tasks, the silence periods were chosen to start 0.5 s after voice offset to avoid overlap with the next trial. This ensured that the periods chosen were during the washout period without other speech-related processing. The HG feature windows were concatenated across electrodes.

To determine whether the information used to decode speech intent was truly a production-related signal, rather than other language-related processes or simply working memory of the word, we also built decoders between speech intent and the middle of the instructed delay period, defined as 0.9–1 s after the word disappeared from the screen (figure 2). We used 4 HG feature windows just prior to each delay or speech period as features in this analysis. The same procedure was applied to extract power and construct feature windows from the alpha (8–12 Hz) and beta (13–30 Hz) bands. To determine whether decoding performance varied based on the task, we analyzed task-based decoding performance using HG features for the three subjects who were cued with both auditory and visual stimuli.

### 2.6. De-mixed principal component analysis (dPCA)

For visualization purposes, we used dPCA [31] to decompose the signals into components that maximized separation between speech intent and silence-related signals. HG power was first smoothed using a centered, 4th^-^order Savitzky Golay filter of 201 ms length. The signals during speech intent and silence periods were treated as two different conditions. In this analysis, the speech intent period was defined as the 500 ms before voice onset, and silence was defined as 1–1.5 s after voice offset. This analysis was performed only on data from participants performing the task that included a go cue to avoid potential confounds from seeing the words onscreen.

### 2.7. Decoding speech intent from all electrodes

Each dataset, comprised of features concatenated across all runs for each participant, was divided into training (80%) and testing (the remaining 20%) subsets. We built support vector machine decoders with radial basis function kernels [32]. Within the training subset, the decoders were 5-fold cross-validated—a decoder was trained on four folds and tested on a held-out validation set. This step was repeated 5 times such that each trial would appear in the validation set at least once. Hyperparameters were optimized by performing a grid search on the penalty term and the shrinkage term of an RBF kernel. The best decoder was identified as the decoder with the highest classification accuracy across 5 folds. The hyperparameters for this decoder were used to train a new decoder on the entire the training subset and tested on the 20% testing subset. This procedure was iterated 20 times, such that in every iteration, the training and testing subsets would be generated by random resampling. The mean test performance across iterations was computed.

To identify the time course of the speech intent-related signal, we built decoders using HG features with varying offsets between the features and voice onset. Offsets of the end of the last feature window in each trial were shifted in 100 ms increments from −1.5 to 0.7 s relative to the onset of speech. To ensure that the decoder was only decoding between speech intent and silence, rather than other processing involved in the tasks, we did not offset the features related to the silence period. We computed the significance of performance accuracy at each offset by comparing it to an empirical chance distribution of testing accuracies with shuffled labels. For each offset, we shuffled the labels of each trial in the testing set and classified each shuffled set. This was repeated 500 times and the testing accuracies from the shuffled testing data formed the empirical chance distribution. At a given offset, a *p*-value was defined as the first point along the empirical distribution at which the mean accuracy across 20 iteration was greater than 95th percentile of the chance distribution. The *p*-values were adjusted for multiple comparisons using the Benjamini–Hochberg false discovery rate correction. To determine significance across participants, an empirical cumulative distribution of the mean shuffled testing accuracies across participants was computed at each offset. The significance at each offset was identified as the percentile in the cumulative across-participant distribution. Accuracy at an offset was considered significant if the mean accuracy across participants was greater than the 95th percentile. All computed *p*-values were corrected for multiple comparisons using the Benjamini–Hochberg false discovery rate.

To identify when the decoding performance first became better than chance, we identified the first offset at which decoders were significant in each participant and computed the mean of these times. Further, to ensure that we included only speech production-related information in our decoders, we computed this average across only the participants who performed tasks with the instructed delay.

It is possible that the above analysis could result in accuracies above chance if the speech intent period contained information about non-vocalization-related, higher-order language processing functions or working memory. For example, in the picture naming task, a participant’s brain had to recognize the picture, associate that with a semantic meaning and a word (lexical selection), then determine the phonological composition of the word to be produced, followed by the speech sounds and articulator movements required to produce that word. Further, they needed to keep the word in working memory during the delay period. Many of these stages have substantial representation in the temporal and/or parietal lobes [33–36]. To control for this possibility, we also decoded between speech intent and the instructed delay period using 4 features. We decoded between speech intent and instructed delay period, again using a range of offsets between features and speech intent windows: from −200 to 0 ms relative to voice onset in 100 ms increments. To ensure a consistent comparison across participants, this analysis was performed on the 5 participants who performed the instructed delay single-word-reading task.

### 2.8. Co-registration of electrodes

For extraoperative participants, electrode locations were identified using the participant’s preoperative MRI and post operative CT. The two images were co-registered using the LeGUI [37] software package for MATLAB. For intraoperative participants, coordinates of the corners of the arrays were registered using BrainLab Curve (in ACPC space) by a neurosurgeon (MCT). The remaining electrodes in each array were interpolated from the registered electrodes. We verified these locations using anatomical markers from photographs taken during the awake surgery. The 3D cortical surface for each intraoperative participant was generated from the preoperative MRI using FreeSurfer [38]. For ensemble electrode visualization across patients, electrodes were transformed into MNI space using LeGUI for the extraoperative participants. Electrodes were manually assigned on the MNI brain based on anatomical markers for the intraoperative participants.

### 2.9. Localizing speech intent information

To assess spatiotemporal patterns of activity related to speech production we performed a cluster analysis. For every participant, we computed the *z*-scored HG power envelope in a time window from 1 s before to 0.5 s after voice onset. We computed the average HG activity across trials for every electrode, then smoothed with a Gaussian kernel of 25 ms width. We clustered activity in these electrodes using an unsupervised clustering technique, *k*-means clustering [39]. We identified the optimal number of clusters as the elbow of the curve depicting the average distance between cluster elements versus number of clusters, varied from 2 to 10 clusters.

Further, to ascertain the areas in the temporal and parietal lobes with the most information about speech intent, we built separate decoders for each individual electrode, again using 4 HG feature windows per trial. We performed this analysis without any temporal offset, decoding between voice onset and silence. We used the same decoding procedures as described above, except only using features from one electrode at a time. Electrodes with significant information about speech intent were identified by comparing the mean accuracy across iterations to a chance distribution as described above, corrected for multiple comparisons using the Benjamini–Hochberg false discovery rate correction. These electrodes were plotted on a standardized brain.

## 3. Results

### 3.1. HG activity increased before voice onset and after go cue onset in some areas

As the temporal and parietal areas play roles in both visual and auditory perception (which we use to include comprehension as well, for brevity), we sought to assess whether they contained causal information regarding speech intent, i.e., preceding voice onset. In many electrodes, HG activity followed voice onset by about 300 ms, suggesting these were responding mainly to auditory perception of the participant’s own speech. However, there was a minority of electrodes (representative examples in figure 3) in which HG activity increased starting about 200–300 ms before voice onset. This further supports the presence of speech intent encoding in the temporal and parietal lobes. Further, this activity appeared time-locked to voice onset, rather than to the go cue (figures 3(a) and (b)). This indicated that the signals were related to speech intent, rather than to perception of the go cue.

### 3.2. Speech intent and silence were separated in a low-dimensional neural subspace

**Figure 4.**
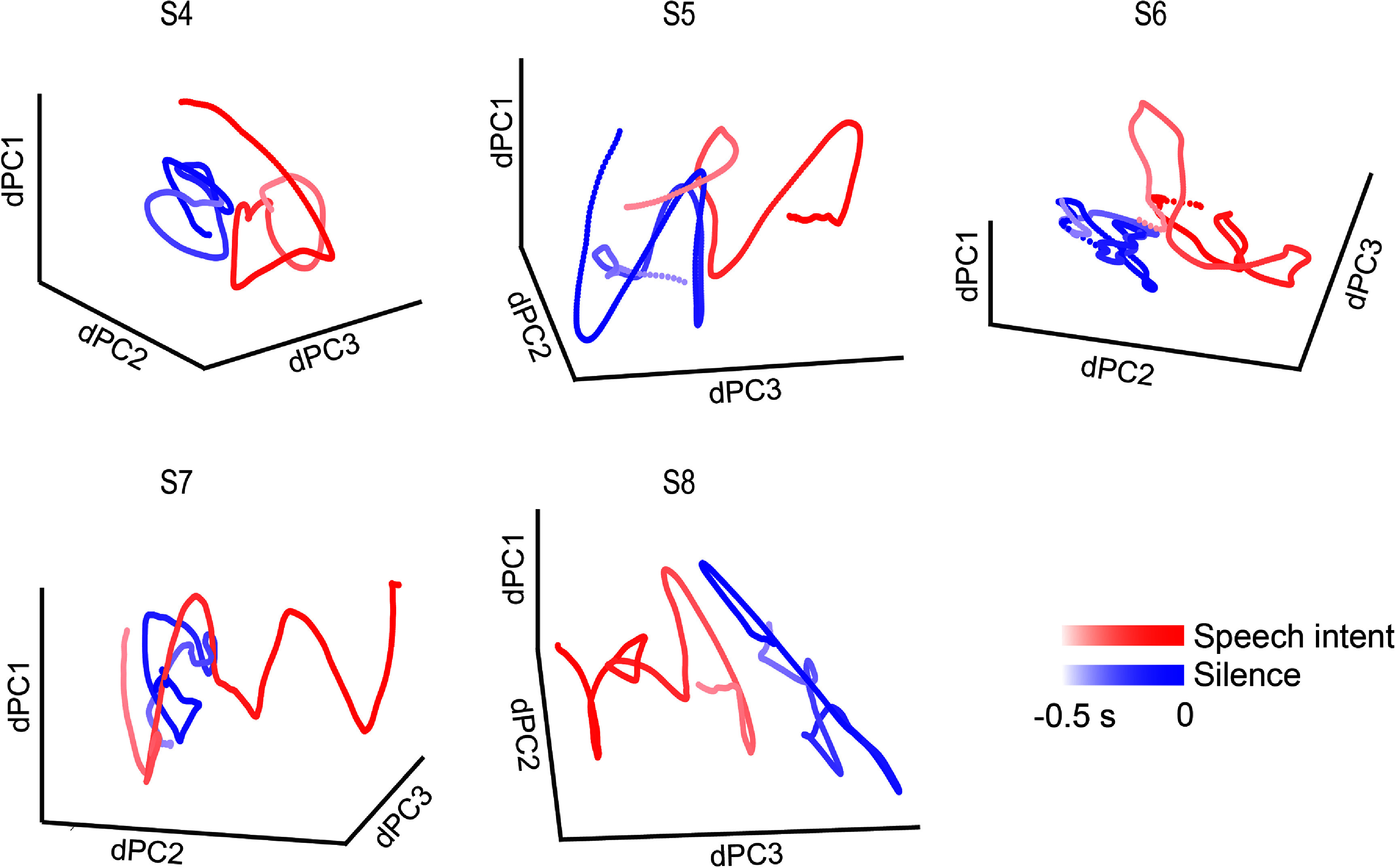
Neural subspace projection in five participants. De-mixed principal component analysis (dPCA) averaged across trials reveals distinct clustering of speech intent (red) and silence (blue) in the state space defined by the first three dPCs. For every trial, speech intent was defined as 500 ms prior to the voice onset up to voice onset. Similarly, the silence period for every trial was defined as a 500-ms window chosen 1.5 s after the voice offset for the current word.

We first sought to determine the extent to which speech intent and not speaking (silence) were separable in neural space. We used dPCA to isolate components of the HG signal related to each condition in 5 of the participants. The first three most significant dPCs accounted for 70.6%–84.5% of variance. The dPC trajectories for each condition were clearly separated (figure 4), especially toward the end of each period (the most saturated part of each curve). This suggested that speech intent could be distinguished from silence using information from the temporal and parietal lobes.

### 3.3. Information in temporal and parietal lobes enabled differentiation of speech intent from silence

**Figure 5.**
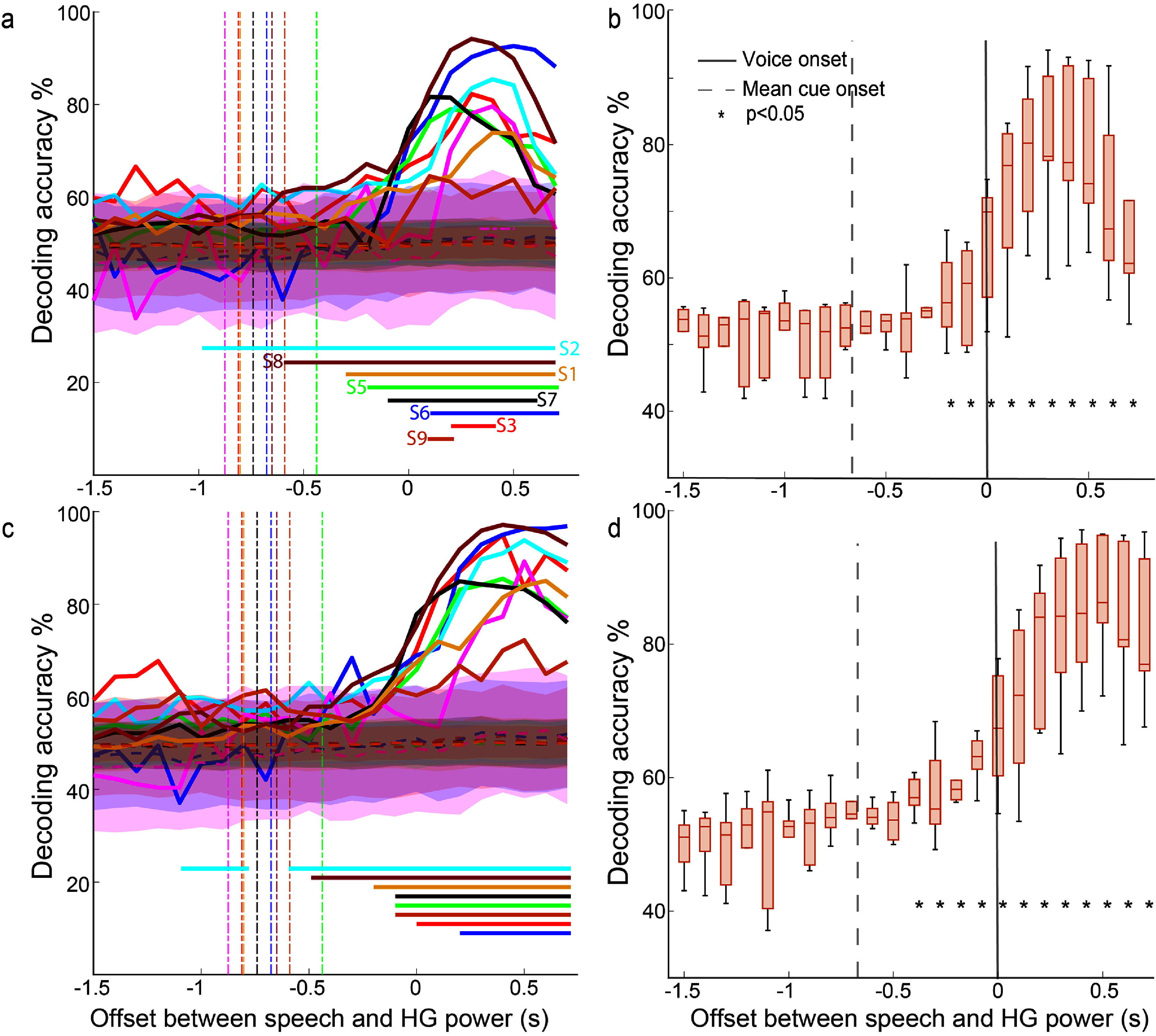
Decoding accuracy at different times for individual and combined participants. (a) Decoding accuracy for each participant (labeled by color) using one feature window at multiple offsets relative to voice onset (0 s). Vertical dashed lines represent the mean onset times of the go cue (or stimulus, for those not performing the instructed delay task) for each participant. Horizontal bars indicate significant values (*p* < 0.05) compared to chance at each offset. (b) Median (±IQR) decoding accuracy across participants at each offset. Error bars denote the range of accuracies. Vertical dashed line represents mean onset time of the go cue. Asterisks indicate significance compared to chance. Vertical solid line represents voice onset. (c) Individual participant decoding accuracies using four 100 ms feature windows at each offset. (d) Median decoding accuracy across participants using four 100 ms feature windows.

We next used temporal and parietal information to decode when participants intended to speak or were silent. First, we used a single, causal HG window prior to voice onset and silence periods. To identify when speech intent signal was present relative to voice onset, we varied the offset between speech periods and HG features as described in Methods. In all participants, discrimination accuracy between speech intent and silence increased as features approached voice onset (figure 5(a)). Maximum accuracy unsurprisingly occurred after voice onset, indicating significant information about perceptive components of speech. We decoded speech intent with accuracies significantly better than chance in 7 out of the 9 participants (all but S4 and S6). The consolidated results across participants (figure 5(b)) revealed that we decoded speech intent better than chance accuracy starting at the bin 200–100 ms prior to the onset to speech. At the bin from 100 to 0 ms prior to voice onset we decoded speech intent with a mean accuracy across participants of 65.3% and range 51.9%–74.8%. On average, across the participants who were cued with an instructed delay, decoding performance became significantly better than chance starting 300 ms prior to voice onset.

We performed the same analysis using 4 consecutive, causal HG windows prior to voice onset and silence periods. Consistent with our single feature window decoding analysis, decoding accuracy steadily increased closer to voice onset in all the participants (figure 5(c)). In eight of nine participants, we decoded speech intent significantly more accurately than chance using information in the 400 ms prior to voice onset. Accuracy rose above chance starting at the window 600–500 ms (range, 0–800 ms across participants) before voice onset (figure 5(d)). At the bin from 100 to 0 ms prior to voice onset, using causal windows, we decoded speech intent with a mean accuracy of 67.5% with range 54.6%–77.8%.

There were two participants (S4 and S6 in the single feature-window analysis; S4 in the four feature-window analysis) for whom we could not decode better than chance using only causal information. Notably, these participants completed fewer than 60 trials as compared to a range of 147–496 trials in the other participants. Furthermore, to validate our methods, we performed the same analysis using only electrodes over the occipital lobe (only participant S5 had this coverage). Classifiers did not perform significantly better than chance using information from the occipital lobe.

### 3.4. Information about speech intent was related to production rather than language processing or working memory

**Figure 6.**
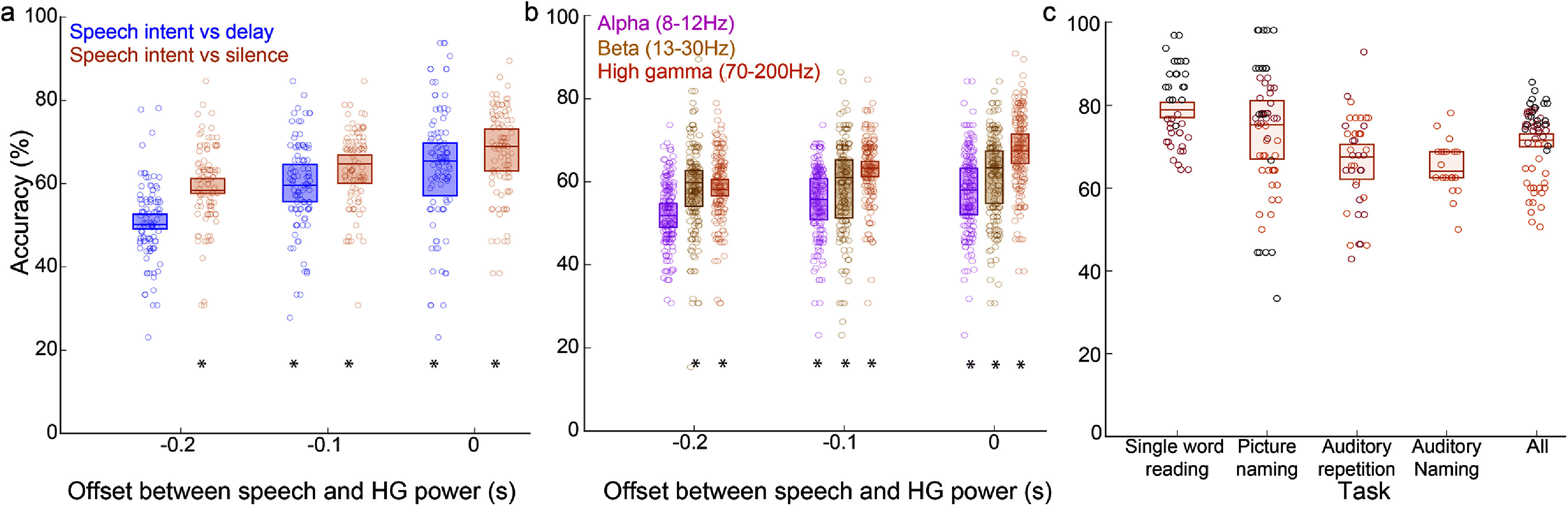
Controlling for language processing, frequency bands and tasks. Boxplots represent median (±IQR) accuracy across participants. For every offset, decoding accuracy over 20 iterations was averaged across participants. This was compared to a chance distribution across participants computed similarly. Each colored dot represents one decoding iteration from one participant. (a) Accuracy across participants of decoding between speech intent and silence (orange) and between speech intent and instructed delay (blue) for the five participants (S4–S8) that performed the single word reading task with an instructed delay period. (b) Decoding accuracy using different frequency band features across all participants. (c) Decoding accuracy for different speech tasks for the three participants (S7–S9) that performed multiple tasks at 0 s offset. * p < 0.05 (FDR corrected) in all panels.

The speech tasks performed in this study required not only production, but also perceptive speech and language processing. To control for these other processes—particularly lexical selection, phonological composition, and working memory—we applied the same decoding method as before, but this time classified between speech intent and instructed delay periods. We analyzed data from the five participants (S4–S8) who performed the single-word reading task with an instructed delay period. We computed this accuracy at three offsets prior to voice onset using causal information prior to voice onset (at 0 s). We found information enabling accurate decoding between speech intent and instructed delay periods. At 0 s offset (i.e., from 100 ms to 0 ms prior to voice onset) we decoded between speech intent and instructed delay periods with an accuracy of 64.33 ± 12.1% (mean ± SD across participants) and decoded between speech intent and silence periods with an accuracy of 67.7 ± 8.5% (figure 6(a)). Accuracy of decoding between speech and delay was not significantly different (*p* = 0.4 at −0.1 s and *p* = 0.4 at 0 s; Wilcoxon rank sum test) from accuracy of decoding between speech and silence for two offsets and was significantly different at −200 ms prior to the onset of speech (*p* = 0.007). This provided further evidence that the temporal and/or parietal cortices contained information about speech intent, rather than only language processing or working memory.

### 3.5. Decoding performance using different frequency bands and tasks

While most ECoG BMIs have used HG band as their primary feature for decoding, some evidence suggests that there may be speech-related information encoded within other frequency bands as well [40]. In addition to high gamma band, we built decoders using either alpha (8–12 Hz) or beta (13–30 Hz) bands. We computed the accuracy across all participants at 3 offsets prior to voice onset (figure 6(b)). At 0 s offset, we decoded between speech intent and resting period with a mean accuracy of 57.7 ± 7.1%, 61.4 ± 7%, 67.5 ± 5.9% using features computed using the alpha, beta, and high gamma bands, respectively. HG band consistently provided the most information, while beta band also provided performance better than chance prior to speech onset.

Three participants performed multiple tasks, including both visual and auditory stimuli. To assess for any differences in speech intent related to modality, we compared decoding performance across tasks for these three participants. This analysis was performed at 0 s offset using causal information prior to voice onset (figure 6(c)). There was somewhat better decoding performance in the single word reading task 79.2 ± 10.8%. However, this average was computed over 2 participants only since S9 did not perform this task.

### 3.6. Spatial localization of speech intent signal

**Figure 7.**
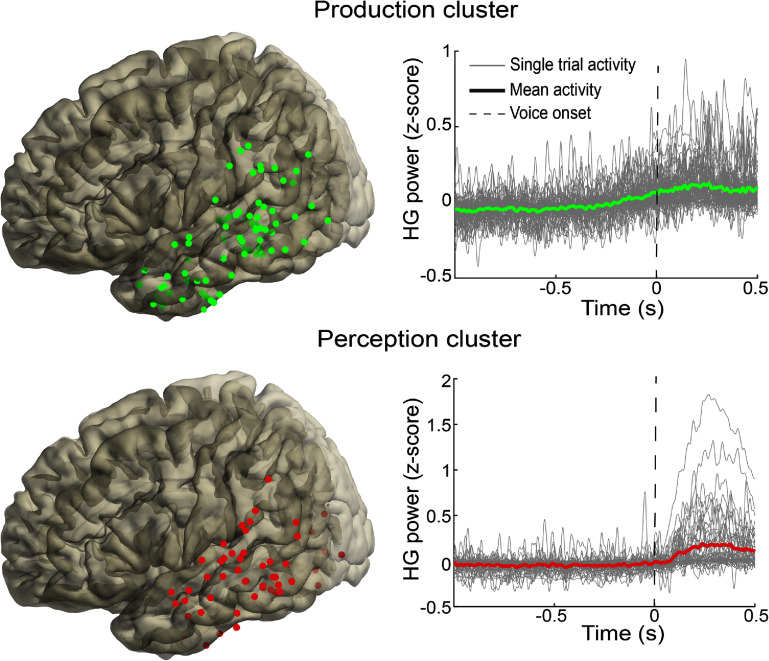
Cluster analysis results. We identified 2 clusters that modulated with speech. Right, two clusters of activity related to production (top, green) and perception (bottom, red). Left, distribution of the electrodes in each cluster shows widely distributed pattern across the temporal and parietal lobes.

To identify areas that tended to modulate similarly with respect to voice onset, we performed k-means clustering on the mean HG power time courses. We identified five clusters as the optimal number. Two clusters showed noticeable modulation with respect to voice onset (figure 7). In one cluster, which we named the production cluster, mean activity within the cluster showed an increase in power modulation starting approximately 300 ms prior to voice onset. A second cluster contained perceptive speech-related information: an increase in mean activity starting approximately 100 ms after voice onset, indicating activity related to the participant hearing themselves talk. Activity in these clusters was distributed widely across parietal and temporal lobes. In the production cluster, electrodes were localized somewhat more densely in posterior middle and inferior temporal gyri, with sparser representation in anterior temporal lobe and supramarginal gyrus. An important caveat of this clustering method is that k-means is a hard clustering technique, which means it was possible that an electrode modulating with both production and auditory perception was assigned to only one cluster.

**Figure 8.**
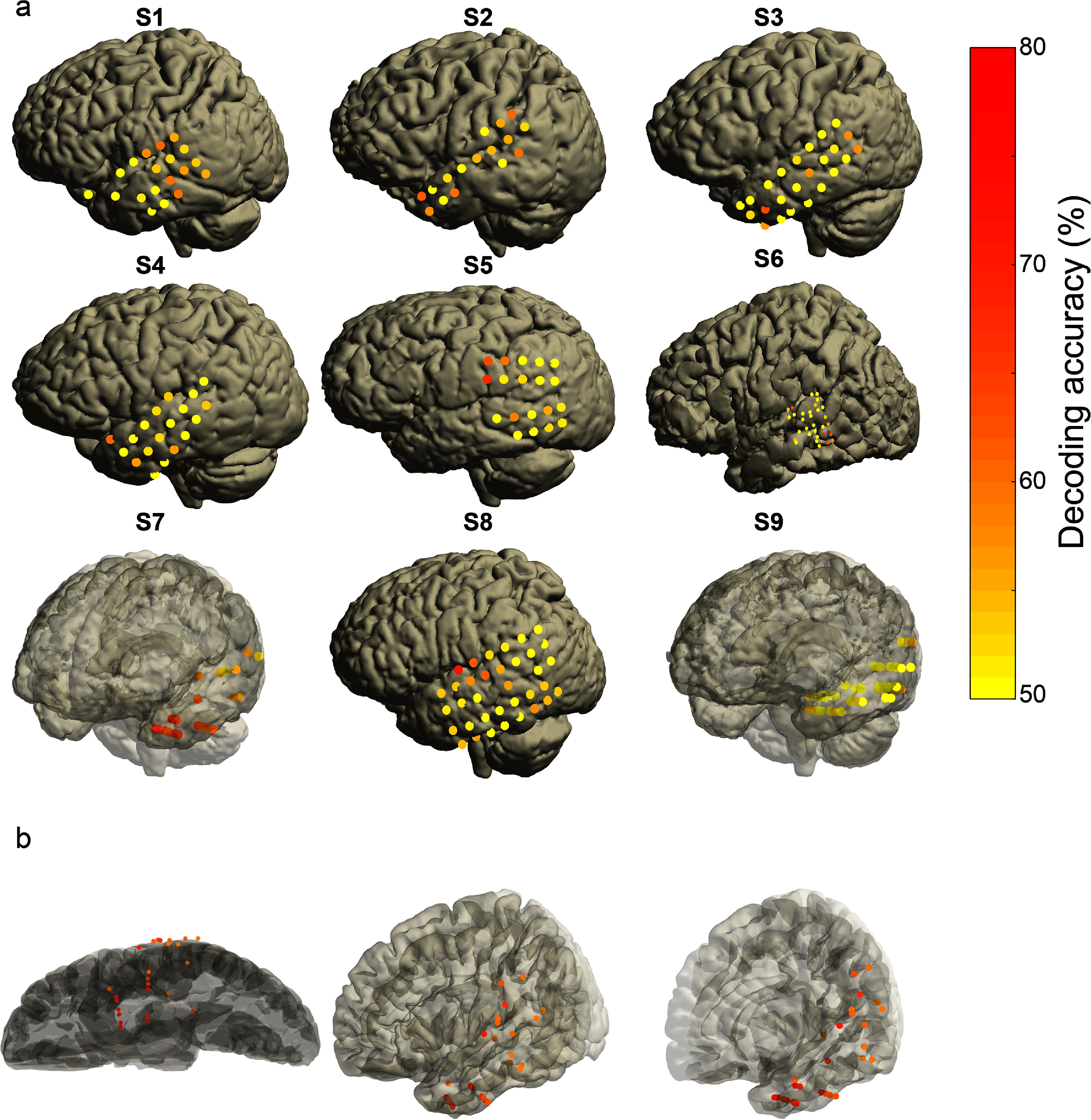
Speech intent decoding accuracy on individual electrodes before voice onset. (a) Individual participants; electrode color denotes decoding accuracy. (b) Speech intent decoding with only the significant electrodes, across all participants, plotted on an MNI brain in different orientations.

To further assess the location of the speech intent signal, we used single electrodes to decode speech intent from silence. Information about speech intent was spread heterogeneously across participants (figure 8). In some participants, information was distributed rather evenly (e.g. S1, S3) while for others it was more tightly clustered (e.g. S6). The greatest amount of information appeared to be in the anterior superior temporal (S1, S3, S4, S6, S8), middle temporal (S6), and supramarginal (S5) gyri, as well as primary somatosensory cortex (S2). When viewing all electrodes with decoding accuracy greater than chance (*n* = 27) in MNI space (figure 8(b)), the significant information was distributed across multiple areas, including the posterior middle and superior temporal gyri, posterior parietal cortex (especially supramarginal gyrus), and anterior inferior temporal gyrus.

## 4. Discussion

BMIs for speech production have made impressive progress, yet might also benefit from using signals from temporal and parietal cortices. However, because these cortices are involved in perceptive speech processing, it is critical to be able to distinguish speech intent from perceptive speech or thoughts unintended to be voiced aloud. In this study, we showed evidence of causally timed information about intent to voice speech in parietal and temporal cortices. Neural signals related to speech intent occupied a low-dimensional subspace distinct from those during silence periods. This information enabled accurate decoding between speech intent and silence starting approximately 200–300 ms prior to voice onset. Further, this information also enabled decoding between speech intent and an instructed delay period, indicating that this truly was related to motor intent, rather than higher-order language processing or working memory. Some information about speech intent was also seen in the beta band. We found slightly higher decoding performance when the participants were cued visually, especially in the single-word reading task. However, multiple other variables could explain this difference, such as differences across individual brains and electrode numbers and coverage, signal quality, the number of trials performed, and the number of subjects that performed each task. We did not see a substantial difference in performance between different electrode types- 66.8 ± 5.5%, 68.9% and 69.0 ± 4.8% for the participants with clinical grids (6 participants), high density grids (1 participant) and sEEG electrodes (2 participants) respectively at voice onset. Finally, the information was distributed through much of the lateral temporal and parietal cortices with a somewhat greater concentration in superior temporal, middle temporal and supramarginal gyri.

Prior studies have found some information about speech in temporal and parietal lobes. Lesion and fMRI studies have indicated that the STG may be important for phonological processing [41] and semantics [17, 33]. Parietal areas have also been implicated in lexico-semantic encoding [42, 43]. These areas are also strongly involved with speech perception and comprehension [20, 44]. However, fMRI does not have sufficient temporal resolution to resolve differences between speech production-related and speech perception-related processing. Further, intracranial recordings have shown to encode several aspects of speech. STG represented phoneme features (manner of articulation) during an auditory perception task [20]. Supramarginal gyrus recordings during a speech production task contained some semantic information [25, 26]. ECoG recordings from a broad network including frontal, temporal, and parietal cortices enabled decoding of sentences with a deep learning and language model [28]. Only two of these studies made some distinction between information related to auditory perception and speech production. However, the involvement of the temporal and parietal lobes in intention to talk, specifically motor intent, has not been investigated.

### 4.1. Scientific implications of speech intent information outside of frontal lobe

Typically, we think of motor signals in frontal areas, with some higher-level planning in posterior parietal areas [45, 46]. However, speech production is a somewhat unique motor function since it involves both cognitive and motor components. Here, we found that speech intent was decodable starting at about 300 ms before voice onset using information from both the temporal and parietal cortices. Prior studies showed that HG power starts to increase 100–300 ms before voice onset in M1 [47] and 200–400 ms before voice onset in temporal cortices (STG and fusiform gyrus) during a single-word reading-aloud task without instructed delay [48]. The timing of the speech intent-related information in our study and the ability to discriminate between intent and the instructed delay period indicate that the speech intent signal is indeed related to vocal motor production. The intent-related information was highest in STG, middle temporal gyrus, angular gyrus and supramarginal gyrus, but overall was distributed rather widely. The origin of this speech intent signal is as yet uncertain.

Forward models of speech production propose the encoding of an auditory goal analogous to a motor goal within the auditory cortex [49, 50]. They hypothesize that during speech production an efference copy of the motor signal is utilized within the auditory cortex to compare the motor output against the audio input [51, 52]. Behavioral evidence suggests compensatory changes in speech are observed when auditory feedback is manipulated [53–55]. Further evidence for efference copy was found in ECoG and magnetoencephalographic studies showing inhibition in STG due to participants hearing themselves talk [51, 56]. Our results could be related to an efference copy signal. However, the wide distribution of intent-related activation across the temporal and parietal lobes, including to non-acoustically related areas not previously described as receiving efference copy signals, suggests that this information may be more than an efference copy signal.

Another possibility is that the intent signal is initiated locally in temporal and/or parietal areas. To our knowledge, evidence for an intent signal in temporal or parietal areas has not been previously described. However, data from some prior ECoG studies have shown hints of increasing HG power in temporal areas during speech production, though the studies’ tasks were not designed to disentangle perceptive language and produced speech [2, 48, 57, 58]. Also, nonfluent (Broca’s) aphasia is classically thought to be caused by damage to the frontal lobe. The intent to speak is still present in such patients, suggesting it is generated outside frontal areas. A recent study of surgical resections showed that supramarginal gyrus (including underlying white matter) may also play a bigger role in Broca’s aphasia [59], suggesting that parietal areas, as well as connections from other temporal or parietal areas, are directly involved in speech production. Further studies are needed to determine the precise origin of the speech intent signal.

### 4.2. Implications for BMIs

To date, speech BMIs have primarily targeted people with locked-in syndrome, in whom the motor cortices are intact but downstream connections are broken. Information about speech intent from non-frontal areas, combined with other information about speech [17, 24, 26, 28], could improve BMI design. In the case of locked-in patients, temporal and parietal signals could add more, and different types, of information than frontal areas contain. Additionally, people with amyotrophic lateral sclerosis often have degeneration of motor cortical neurons, as well as spinal motoneurons, so information from other areas might enable longer-term, or a broader patient eligibility for, use of BMIs. Moreover, a much larger population of individuals with frontal lobe damage from stroke, brain trauma, or tumors causing nonfluent (a.k.a. expressive or Broca’s) aphasia or apraxia of speech, who might benefit from a BMI to decode intended speech. These syndromes are caused largely by damage to ventral motor/premotor cortex (apraxia of speech [60, 61]) and to IFGs (Broca’s area) plus underlying white matter connections, as well as to supramarginal gyrus [59]. Such aphasic patients have agrammatic and sparse speech but usually retain at least some direct control over their articulators (though there may be co-existing apraxia of speech), and often show frustration due to not being able to express their thoughts. In such patients, the intended meaning is present. However, since IFG and precentral gyrus are often damaged in such patients (e.g., after a middle cerebral artery stroke), these areas are not likely to be available to provide input to a BMI. Such individuals represent a much larger population than those with locked-in syndrome, so this might broaden the population of patients who could benefit from a communication BMI. To use signals from these areas, it would be essential to know when a person is trying to speak, because decoding thoughts would be unethical. Establishing the existence of a speech intent signal that can be decoded from non-frontal cortical areas is therefore critical to design of a speech BMI for such individuals.

## Data Availability

The data cannot be made publicly available upon publication because they contain sensitive personal information. The data that support the findings of this study are available upon reasonable request from the authors.
